# Preliminary Experience with Three Alternative Motion Sensors for 0.55 Tesla MR Imaging

**DOI:** 10.3390/s24123710

**Published:** 2024-06-07

**Authors:** Radhika Tibrewala, Douglas Brantner, Ryan Brown, Leanna Pancoast, Mahesh Keerthivasan, Mary Bruno, Kai Tobias Block, Bruno Madore, Daniel K. Sodickson, Christopher M. Collins

**Affiliations:** 1 Bernard and Irene Schwartz Center for Biomedical Imaging, Department of Radiology, New York University Grossman School of Medicine, New York, NY 10016, USA; 2 Center for Advanced Imaging Innovation and Research (CAI^2^R), Department of Radiology, New York University Grossman School of Medicine, New York, NY 10016, USA; 3 Vilcek Institute of Graduate Biomedical Sciences, New York University Grossman School of Medicine, New York, NY 10016, USA; 4 Siemens Medical Solutions USA Inc., New York, NY 10016, USA; 5 Department of Radiology, Brigham and Women’s Hospital, Harvard Medical School, Boston, MA 02115, USA

**Keywords:** sensors, MRI, motion, therapy, low-field, ultrasound, Pilot-Tone, Time-of-Flight

## Abstract

Due to limitations in current motion tracking technologies and increasing interest in alternative sensors for motion tracking both inside and outside the MRI system, in this study we share our preliminary experience with three alternative sensors utilizing diverse technologies and interactions with tissue to monitor motion of the body surface, respiratory-related motion of major organs, and non-respiratory motion of deep-seated organs. These consist of (1) a Pilot-Tone RF transmitter combined with deep learning algorithms for tracking liver motion, (2) a single-channel ultrasound transducer with deep learning for monitoring bladder motion, and (3) a 3D Time-of-Flight camera for observing the motion of the anterior torso surface. Additionally, we demonstrate the capability of these sensors to simultaneously capture motion data outside the MRI environment, which is particularly relevant for procedures like radiation therapy, where motion status could be related to previously characterized cyclical anatomical data. Our findings indicate that the ultrasound sensor can track motion in deep-seated organs (bladder) as well as respiratory-related motion. The Time-of-Flight camera offers ease of interpretation and performs well in detecting surface motion (respiration). The Pilot-Tone demonstrates efficacy in tracking bulk respiratory motion and motion of major organs (liver). Simultaneous use of all three sensors could provide complementary motion information outside the MRI bore, providing potential value for motion tracking during position-sensitive treatments such as radiation therapy.

## 1. Introduction

The need to limit well-documented classes of motion artifacts in magnetic resonance imaging (MRI) has resulted in the development of a wide range of motion correction techniques to minimize image distortions and enhance robustness. Excessive motion-related degradation of image quality can render MRI scans non-diagnostic and can lead to reduced scan efficiency and increased costs [1,2,3]. Various types of motion, including bulk, respiratory, and cardiac motion, have been identified during scanning, each requiring distinct correction strategies. With the growing interest in imaging at lower field strengths to enhance MRI accessibility and cost-effectiveness, the relevance and complexity of motion correction are amplified [4,5]. Due to the signal-to-noise ratio (SNR) diminution inherent in low-field MRI, longer scan times are required, which exacerbates both patient discomfort and susceptibility to motion artifacts [6].

Numerous motion correction devices and strategies have been developed and validated for diverse use cases, for example, MR navigator pulses [7], respiratory belts [8], and MR spirometry [9]. However, these methodologies present inherent limitations. For example, navigator-based strategies require explicit pulse sequence programming, advanced reconstruction techniques, and often fat saturation pulses. This limits their application to specific sequences, and entails additional data acquisition, which can further elongate already protracted imaging times at lower field strengths [10,11]. In addition, although respiratory belts are commonly used for tracking respiratory motion, they offer no insight into other internal motion [8].

Recently, two new sensors have emerged to address some of these limitations. One is a small ultrasound sensor, also called an Organ Configuration Motion (OCM) sensor [12,13], and the other is the Pilot-Tone, a radiofrequency transmitter [14,15,16]. Both have shown effectiveness in compensating for respiratory and cardiac motion, suggesting potential for tracking internal organ motion. Low-field MR imaging allows easier use of electronic devices (such as these sensors) by minimizing potential interference between the MR fields and sensor acquisition apparatus. Additionally, safety concerns are reduced due to the lower RF power and main magnetic field strength. However, the performance and technical set-up of these new sensors in low-field MR systems remain to be evaluated.

In this paper, we investigate the use of three MRI-compatible sensors and demonstrate their efficacy in tracking various types of motion at 0.55 T. These sensors consist of the Pilot-Tone (using RF electromagnetic energy) for monitoring respiratory and liver motion, the OCM sensor (using ultrasonic mechanical vibrations) for tracking bladder motion, and a 3D Time-of-Flight (ToF) camera (using near-infrared light) for tracking bulk motion. Furthermore, we demonstrate their capability to track motion independent of MRI, offering avenues for compensating internal and external motion during procedures conducted outside the MRI bore, such as radiation therapy, where estimated motion states can be used to correlate to previously measured time-dependent anatomical configurations [17].

## 2. Materials and Methods

**Figure 1:**
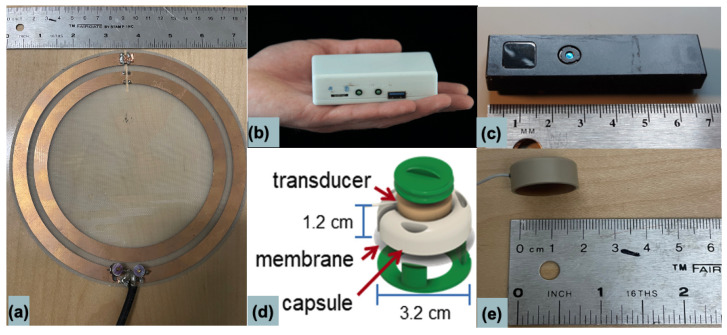
The sensors used in this study. (**a**,**b**) The Pilot-Tone (PT), typically placed near the MR bore, is a radiofrequency transmitter that induces motion-related modulations in the signal received through the RF coils during MRI. (**a**) shows the PT in its loop antenna form used in this study at 0.55 T and (**b**) shows the PT in its compact, self-contained form distributed for use at 3 T. (**c**) The Time-of-Flight camera uses pulses of near-infrared light to measure the distance between the camera and all surfaces in its field of view, such as the abdomen, during respiration. (**d**,**e**) The single-element OCM ultrasound sensor, when adhered to the patient, is sensitive to a variety of different motions, depending on its placement. (**d**) shows a diagrammatic representation of the OCM sensor when contained in its capsule; (**e**) shows the OCM sensor as used in this study (no capsule used).

The three sensor devices are shown in Figure 1. In this section, we describe the use of each sensor individually, followed by a subsection on their simultaneous use outside the MRI bore to demonstrate their complementary behavior. All MR acquisitions mentioned in this section were performed with informed consent on volunteers recruited as part of an IRB-approved study on a commercial 1.5 T MRI system (MAGNETOM Aera; Siemens Healthcare, Erlangen, Germany) modified to operate at 0.55 T with 23.59 MHz center frequency.

### 2.1. Pilot-Tone

#### 2.1.1. Description and Technical Principle

The Pilot-Tone (PT) is an independent radiofrequency (RF) transmitter placed near the MR bore. Physiological movements, such as respiration, cause variations in the loading of the MRI receive coils, resulting in a modulation of the PT signal received by the MRI RF coils [18,19,20,21]. These modulations, tracked over time, can be used to extract a respiratory signal using signal processing (described in the following sections), while these transmitters are available in compact self-contained units for use at 3 T (Figure 1b), for this study at 0.55 T, the PT signals were generated using an Agilent E4420b RF generator to drive a loop antenna (Figure 1a) placed outside the MRI bore. To avoid interference between the PT and the MR signal, there was an offset (typically 100–250 kHz) between the center frequency of the MRI system and that transmitted by the PT [14].

#### 2.1.2. MRI and Pilot-Tone Data Acquisition

In this study, we evaluated use of the Pilot-Tone for monitoring respiratory motion in liver imaging. Three healthy male volunteers were recruited for this study. Volunteers were instructed to breathe normally for most of the acquisition, except for 1–2 coughs and deep breaths. Images were acquired using two MR sequences: a calibration sequence and a testing sequence. The calibration sequence was used to track motion and train the deep learning model (Section 2.1.3) and the testing sequence was used to demonstrate if any motion correction can be performed when ground-truth respiratory data are not available using the MRI. The calibration was performed using a single-slice 2D bSSFP sequence with high temporal resolution (frame rate = 17.69 frames/s) and the following parameters: TR = 2.26 ms, TE = 1.13 ms, matrix size = 128 × 128, in-plane resolution = 2.34 mm × 2.34 mm, pixel bandwidth = 1955 Hz/pixel, slice thickness = 8 mm, and a phased array body coil. The testing acquisition was a 3D stack-of-stars spoiled GRE sequence (RAVE) with the following parameters: TR = 3.57 ms, TE = 1.69 ms, matrix size = 256 × 256, in-plane resolution = 1.56 mm × 1.56 mm, pixel bandwidth = 500 Hz/pixel, slice thickness = 5 mm, and a phased array body coil.

During MR imaging, concurrent PT data were acquired at two different transmit frequencies: 23.81 MHz for the 2D bSSFP calibration sequence and 23.69 MHz for the 3D RAVE testing sequence. In both cases, spectral overlap was avoided by ensuring that the PT frequency fell outside of the imaging bandwidth, as dictated by the sequence parameters and the center frequency of the scanner [14]. For all scans, the raw data and the inline reconstructed images were exported.

#### 2.1.3. MRI and Pilot-Tone Data Processing and Deep Learning for Liver Position Prediction and Motion Correction

Raw sensor data processing: Since the PT signal is received simultaneously with the MRI signal, it needs to be separated from the raw MRI data and processed. Following a Fourier transformation along the readout direction, the PT signal contribution appears as a visible peak in the spectrum in each line of k-space. An existing PT detection algorithm [14] was used to extract the received PT signal. This signal was extracted and saved for all the calibration sequences as well as the testing sequences on all volunteers to be used in the deep learning model.

Image processing: In this study, the liver was chosen as a target organ for position tracking during respiratory motion. For automatically segmenting the liver, a 2D U-Net was trained to identify the upper liver adjacent to the lungs in order to directly track liver motion in 2D bSSFP images [22]. The network was trained using a Dice loss function, an Adam optimizer, learning rate of 1 × 10^−4^, and batch size of 10. This was possible since the bSSFP sequence results in many 2D slices over time for each patient, with variability in the liver position as the subject breathes. This provided enough data to train the 2D UNet by treating each slice as a separate training instance. A 70–20–10% split was used for training–testing–validation. During inference on the test patient, the segmentation masks from the network were automatically post-processed using the Python SciPy library [23]. Two post-processing steps were performed: (1) a fill-holes algorithm was used to identify and fill all enclosed, non-foreground regions within the target liver binary mask to create a solid, contiguous foreground area by using a 2 × 2 round structuring element; and (2) a 3D connected component method was used to identify and isolate the single largest contiguous region of interest within the liver binary mask by labeling all connected regions, calculating their sizes, and retaining only the largest one (which corresponds to the liver). These steps were used in order to rectify any minor errors made in predictions by the segmentation network (i.e., any stray pixels or islands). The Dice similarity scores were calculated on the test set to show reliability of the predicted segmentation masks. After predicting the liver–lung boundary using the network, a motion curve was extracted for each subject by identifying the median pixel location along the boundary.

Deep Learning Motion Model training: Using the same data split as in the segmentation model training, a fully connected network (FCN) was used in order to predict the superior–inferior displacement of the liver using the PT signal as input [24]. The network contained 5 fully connected layers (100 elements each), followed by a ReLu activation layer. To train the network, a mean squared error loss function, an Adam Optimizer, and learning rate of 0.001 were used. To train the network, the extracted PT signal curve and the image-based liver displacement curve were used as input and prediction, respectively. Training this network allows the unscaled Pilot-Tone signal to be converted into physical displacement of the target organ for each patient, enabling extraction of tissue- and subject-specific motion curves. During inference mode, the PT signal curve was used to predict the superior–inferior displacement of the liver during the MR scan.

Motion Correction in images: As an additional experiment, the trained motion model was then used to predict the liver motion curve, using the PT signal, during a 6 min 3D RAVE sequence for which no image-based motion data are available. We showed the effect of removing k-space lines associated with aberrant motion in two schemes: (1) removing k-space lines that correspond to more than 20 mm of motion, and (2) removing k-space lines that correspond to the volunteer coughing. Reconstruction was performed in MATLAB using the Fessler and Sutton NUFFT toolbox [25].

**Figure 2:**
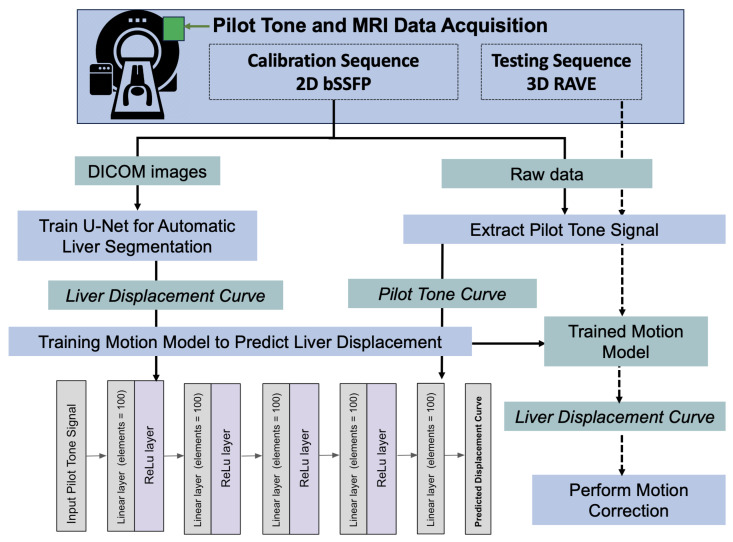
Acquisition and processing of the simultaneous Pilot-Tone and MR data. The Pilot-Tone transmitter is placed near the MR bore prior to scanning the subject. Data from a fast 2D bSSFP sequence and a 3D RAVE sequence are acquired using a phased array coil on the subject’s upper abdomen. Subsequently, bSSFP images are utilized to train a 2D U-Net for liver segmentation, facilitating extraction of the liver–lung boundary to generate a displacement curve. The bSSFP raw data are processed to extract the Pilot-Tone signal from the MR signal via peak identification in the frequency spectrum. The saved liver displacement curves and the Pilot-Tone signal curve are used in a fully connected neural network to train the motion model. During inference, the 3D RAVE raw data are processed to extract the Pilot-Tone signal curve and predict the liver displacement using the neural network. Motion correction in the 3D RAVE data is performed by eliminating k-space lines that are predicted to correspond to (1) more than 20 mm of motion, or (2) coughing by the volunteer.

All MRI data processing and reconstruction was performed using MATLAB (MathWorks, Natick, MA, USA). All deep learning models were trained using PyTorch (Python 3.6). A schematic of the acquisition and processing of the simultaneous Pilot-Tone and MR data is shown in Figure 2.

### 2.2. Ultrasound Sensor

#### 2.2.1. Description and Technical Principle

The ultrasound sensor (8 mm diameter), also referred to as an Organ Configuration Motion Sensor (OCM) [12], consists of a single, 1 MHz, MR-compatible transducer used to both transmit an ultrasound pulse and receive its reflections from the tissues and tissue interfaces. A pulser–receiver is used to generate voltage pulses to fire the sensor and a digitizer card is used to sample the returning signals. More details on hardware specifics can be found in [12].

The emitted ultrasound pattern from this sensor is not focused and is expected to penetrate and reflect from several tissue interfaces within the abdomen. Depending on the placement of the OCM sensor on the body, it can be sensitive to motion of different types, including cardiac, respiratory, surface, or random motion. From this sensor, A-mode ultrasound signals are acquired at a very high frame rate during the MR acquisition, serving as a signature of the organ configuration, including respiratory state or other organ motion.

The OCM sensor was triggered each TR of the imaging sequence and coordinated with the MR acquisition by using an RF pickup loop trigger placed into the bore of the MR scanner [17]. The ultrasound signal, while received separately from the MR signal, is time-stamped and then synchronized with the corresponding MR signal, as described in [12].

#### 2.2.2. MRI and Ultrasound Data Acquisition

In this study, we investigated use of the OCM sensor for tracking bladder motion in the female pelvis. Four healthy female volunteers were recruited for this study. Volunteers were instructed to breathe normally for the acquisition. The OCM sensor was placed approximately 3 inches directly below the navel of each subject. The sensor was coated with ultrasound gel and affixed to the subject using an adhesive bandage. The volunteers were instructed to occasionally tighten their pelvic floor muscles to test the ability of the sensor to detect such motion separate from respiratory motion.

Images obtained with a calibration sequence were correlated with the ultrasound data. The calibration sequence was a single-slice 2D bSSFP acquisition with high temporal resolution (frame rate = 17.69 frames/s) and the following parameters: TR = 2.26 ms, TE = 1.13 ms, matrix size = 128 × 128, in-plane resolution = 2.34 mm × 2.34 mm, pixel bandwidth = 1955 Hz/pixel, slice thickness = 8 mm, and a phased array body coil.

For all scans, the reconstructed images and the raw ultrasound data were exported and stored.

#### 2.2.3. MRI and Ultrasound Data Processing and Deep Learning Prediction of Bladder Position

Image processing: On the reconstructed bSSFP images, Otsu’s method and 3D connected component analysis were used to threshold the images to segment the bladder and calculate its area on the mid-sagittal plane throughout the acquisition. Here, bladder area is used as a measure of internal organ motion, since pelvic floor contraction causes bladder deformation during acquisition.

Raw sensor data processing: The OCM sensor signal was reconstructed as a complex 2D signal as described by Madore et al. [12], where the vertical axis is a surrogate for depth through the body acquired after each US pulse and the horizontal axis is time through the scan. For each time point, the magnitude of the ultrasound signal, representing ultrasound echoes through tissue depth, was used as an input to the neural network. The signal was normalized using its maximum value. This was done in order to use the depth information provided by the ultrasound signal to predict the state (area) of an organ (bladder) from which it is reflected.

Deep learning motion model training: A 1D convolutional neural network (CNN) was trained to see if the ultrasound signal can be used to predict the bladder area during acquisition on an unseen test subject. The network was trained with an AdamW optimizer (learning-rate = 0.0001, weight-decay = 0.0005) and the Huber loss function. Early stopping and weight decay were used to combat over-fitting of the network. During inference, the ultrasound signal from a separate test volunteer was fed into the network to predict bladder area during the entire MRI scan.

**Figure 3:**
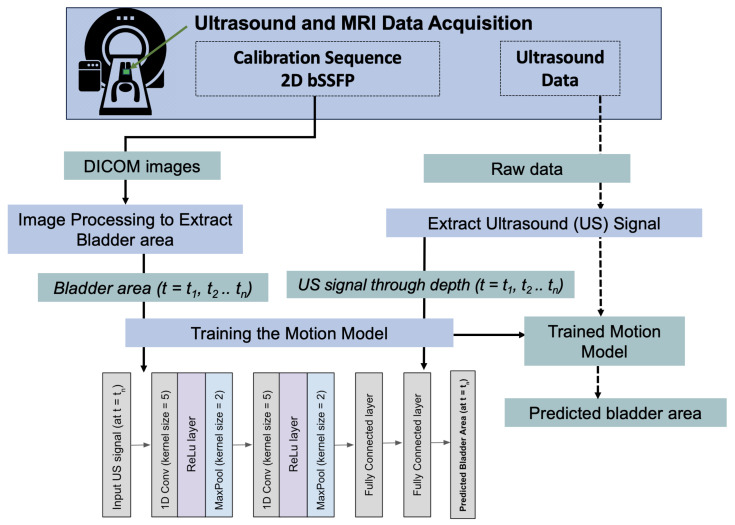
Acquisition and processing of the simultaneous ultrasound and MRI data. The OCM sensor, with ultrasound gel, is positioned on the lower abdomen of the subject before positioning an MRI receive array coil on top of the sensor. The ultrasound acquisition is synchronized with a fast 2D bSSFP sequence via an external single loop of wire placed in the MR bore to detect the RF pulse throughout the MRI sequence. The bSSFP images undergo image processing to extract the bladder and obtain the bladder area over time. The ultrasound raw data are processed to extract the ultrasound signal through depth at each time point corresponding to the bSSFP sequence frames. The saved area trajectory over time and the ultrasound data are used in a 1D convolutional neural network to train the motion model. During inference, the ultrasound is used to predict the bladder area curve using the neural network on an unseen subject.

Raw sensor data processing was performed using SciLab [12]. All deep learning models were trained using PyTorch (Python 3.6). A schematic of the acquisition and processing of the simultaneous ultrasound and MR data is shown in Figure 3.

### 2.3. Time-of-Flight Camera

#### 2.3.1. Description and Technical Principle

A Pico-Flexx 3D Time-of-Flight (ToF) camera (PMD; Siegen, Germany) was used in this study. The camera has dimensions 68 × 17 × 7.35 mm. The ToF camera measures distance to each location on the surface in front of it by measuring the round-trip travel time for bursts of 850 nm (near-infrared) light [26]. Therefore, for measuring respiration, the camera can be mounted on a surface above the subject’s abdomen and is able to track the distance between the camera and the abdomen as it moves during respiration. Using signal processing, a respiratory waveform can then be extracted.

#### 2.3.2. MRI and ToF Data Acquisition

To ensure compatibility of the ToF camera and the MRI system, a phantom experiment was performed before a volunteer exam in order to evaluate any effect the camera might have on MR image SNR. A cylindrical phantom was used and placed in the MR bore for imaging with a phased array coil. The camera was connected to a 25-meter USB cable with the other end connected to a grounded and shielded USB connector fitted in the magnet room filter panel. To minimize interference between the MR system and the camera, the camera was inserted into an 11 cm length of 0.75-inch-diameter copper pipe and the USB cable was passed through a braided copper sheath (TechFlex; Sparta, NJ, USA) that was securely connected to both the copper pipe and the filter panel with copper tape. The copper pipe had a 1.9 × 3.5 cm rectangular window cut into it and a 0.7 mm thick piece of glass coated with an electrically conductive material (Indium-doped tin oxide, or ITO) was cut to fit the rectangular hole in the pipe and affixed to the pipe along all edges with copper tape [27]. The copper pipe was fixed to the inner surface at the top of the magnet bore using adhesive tape at a location directly above the phantom. The ToF data were recorded using the PMD Royale Software version 4.6.0.158 on a laptop computer in the MR console room connected by a short USB cable to the filter panel. The camera exposure was adjusted manually (in software) to minimize the number of overexposed pixels where the distance cannot be discerned. MR image SNR was measured with and without the ToF camera operating in the MR bore during imaging. A two-sided, independent *t*-test, using a significance threshold set at *alpha* = 0.05 was performed on pixel-wise SNR maps within the ROIs to identify any significant difference in the SNR with and without the TOF camera. To measure SNR, a 2D gradient echo sequence with the following parameters was used: TE = 5 ms, TR = 500 ms, flip angle = 25 degrees, FOV = 256 mm × 256 mm, matrix size = 128 × 128, and slice thickness = 10 mm. Additionally, to evaluate the effect of changing the receiver bandwidth on the resulting SNR with the camera, the experiment was performed with 19.2 kHz and 192 kHz receiver MR bandwidths. The camera was set to record at a frame rate of 5 frames/s.

To test the camera’s ability to track respiratory motion, two feasibility experiments were performed on two male volunteers. With the volunteers in the MR bore, the camera was mounted in the bore directly above the abdomen. The camera frame rate was set to 5 frames/s for recording the ToF data. To discern the camera’s ability to resolve distances when objects are placed on top of the subject (typically a coil and/or blanket is placed on a subject in the MR scanner), a body coil with and without a blanket was placed on the subject’s abdomen while imaging with the ToF camera.

In all experiments, data from the camera were saved in the manufacturer’s proprietary file format for processing (described in the next section). Reconstructed images were exported from the MR system for subsequent evaluation of the effect of the camera on MR image SNR.

#### 2.3.3. MRI and ToF Data Processing

The PMD Royale library was used to read the proprietary point cloud time-series files from the ToF camera into MATLAB for further processing. For the MR image SNR evaluation, images collected from the MR system were analyzed using an ROI method. The SNR map was visually inspected and the average SNR in the same ROI for both MR images (with and without ToF camera in bore) was calculated as the mean divided by the standard deviation of signal from identical regions in the images.

For the volunteer experiments, post-processing was performed on the data collected from the camera. For each frame in time, the Z component of the 3D point cloud (in the camera’s frame of reference; distance from camera to abdomen) was averaged over the entire camera frame. Pixels where the distance could not be determined were ignored on a frame-by-frame basis.

### 2.4. Scanner-Less Simultaneous Sensor Tracking of Respiratory Motion Outside the MR System

#### 2.4.1. Physical Components and Technical Principle

In order to (1) test the compatibility of the three sensors; (2) discern complementary/overlapping information obtained from all three sensors; and (3) determine if the sensors can be used without the MR scanner, simultaneous acquisition of data from all three sensors was performed outside the MR system.

#### 2.4.2. Data Acquisition

To utilize the Pilot-Tone in the absence of the MR receive chain, a hand-built receive coil tuned to 23.81 MHz was placed on the abdomen of a male volunteer and connected to a low-cost receive-only Software Defined Radio device, RTL-SDR V.3 (rtl-sdr.com, accessed on 1 October 2023 ). The RTL-SDR was connected to a standard laptop via USB cable and the Pilot-Tone signal was recorded to disk in Matlab R2021a using the RTL-SDR support in the Matlab Communications Toolbox. The ultrasound transducer was positioned a few cm below the sternum of the volunteer. Since there was no need to transmit ultrasound pulses synchronous to the MR acquisition, the ultrasound data were acquired without using the RF loop pickup trigger. The Time-of-Flight camera setup did not require any change and was used as described in Section 2.3.2 above. Shortly after acquisition was begun with all three sensors, the volunteer was instructed to take a deep breath, exhale, cough a few times, and then, breathe normally.

#### 2.4.3. Data Processing

The received Pilot-Tone signal was saved as complex (IQ) samples at a 3 MHz sampling rate. The files were loaded into MATLAB and a fast Fourier transform was performed on the complex signal in groups of 5120 consecutive samples. The amplitude of the recorded signal at the Pilot-Tone frequency was taken from the spectrum at each time point, and a 9th-order median filter was applied to smooth the respiratory signal through time. The OCM sensor data were reconstructed as complex data and processed as described previously [12] to obtain displacement-over-time curves extracted using the ultrasound data from two regions of the abdomen (0–3.3 cm and 3.3–6.6 cm). The ToF data were processed as described in Section 2.3.3 above to extract the respiratory displacement curves.

## 3. Results

### 3.1. Pilot-Tone

**Figure 4:**
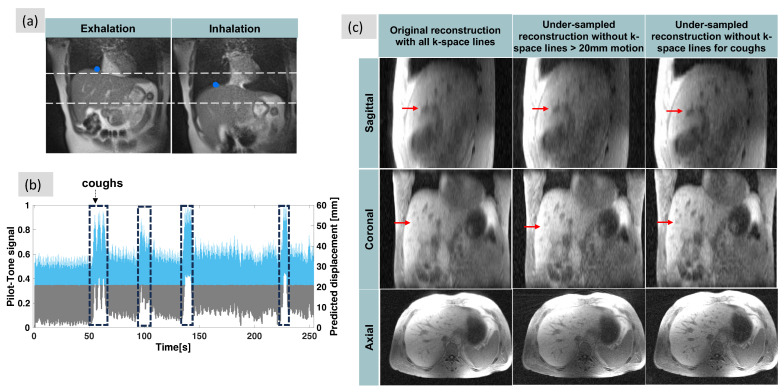
Results of the Pilot-Tone motion experiment. (**a**) bSSFP images showing liver motion during an exhalation and inhalation, and the corresponding extracted liver–lung boundary (blue dot) extracted using the neural network. Significant displacement of the liver is observed during breathing motion. (**b**) The Pilot-Tone signal (arbitrary units, left axis) and the predicted displacement by the trained motion model (mm, right axis) on the 3D RAVE data. A nearly linear scaling is observed between the Pilot-Tone signal curve and the respiration displacement curve. (**c**) Images reconstructed before (left) and after removing k-space lines that were collected during aberrant motion, including when liver displacement was predicted to be 20 mm or larger (center), or when the volunteer was coughing (right). Axial, sagittal, and coronal views are shown. Even though fewer k-space lines are used in the motion-resolved reconstructions than in the uncorrected images, removing extreme motion states results in improved delineation of vessels (red arrows), reduced artifacts within and at the boundary of the liver and other organs, and increased contrast.

The Dice similarity score on the test set for the automatic liver segmentation was 0.93. The mean squared error for predicting the superior–inferior displacement of the liver using the Pilot-Tone curve as input on the test set was 0.013. Figure 4a shows the liver displacement during an exhalation and inhalation, and the corresponding extracted liver–lung boundary (blue dot) extracted using the neural network. Figure 4b shows the derived Pilot-Tone signal (left axis) and the motion curve predicted by the trained motion model neural network using the Pilot-Tone curve as input (right axis) on the 3D RAVE data. As a preliminary demonstration, Figure 4c juxtaposes images reconstructed from all k-space lines with motion-corrected images obtained by eliminating k-space lines corresponding to more than 20 mm of motion (center) or coughs by the volunteer (right). Reduced streaking and blurring artifacts, clearer boundaries, and sharper vessels are observed after motion correction. These changes are more noticeable in the cough-motion-resolved reconstruction compared to the 20-mm-motion-resolved reconstruction.

### 3.2. Ultrasound

**Figure 5:**
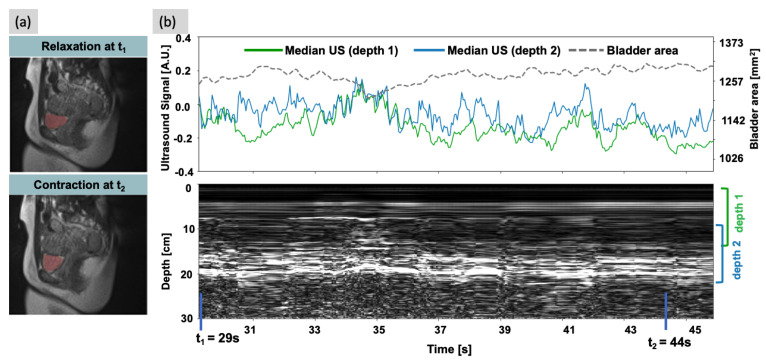
Results of the ultrasound motion experiment. (**a**) Bladder segmentation masks on the fast bSSFP sequence images with normal relaxed muscles (top) and pelvic floor contraction/squeezing (bottom) obtained using image processing. It can be seen that the bladder changes shape as the pelvic floor is contracted, causing a reduction in its area in the mid−sagittal plane. (**b**) 1D plots (top panel) of the median ultrasound signal taken within two different depths from the magnitude of the complex−reconstructed ultrasound data (bottom panel). In the 2D ultrasound plot, the vertical axis represents the approximate depth through the body. The horizontal time axis is coordinated with the fast bSSFP sequence to observe changes in bladder area (dashed curve, top panel) and corresponding ultrasound signals. The correlation between the ultrasound signal and the observed bladder area is not linear.

Figure 5a shows an example of the segmented bladder on the bSSFP sequence at two time points (t_1_ and t_2_, also marked on Figure 5b), without and during pelvic floor contraction, showing changes in bladder shape. Figure 5b (bottom panel) shows the absolute magnitude of the complex ultrasound signal over time. The median ultrasound signal at the two depths, along with the bladder area calculated from the bSSFP images, are shown in the top panel of Figure 5b. The mean absolute error of the prediction of bladder area on the test subject using the trained 1D CNN is 16.4 mm^2^.

### 3.3. Time-of-Flight (ToF) Camera

**Figure 6:**
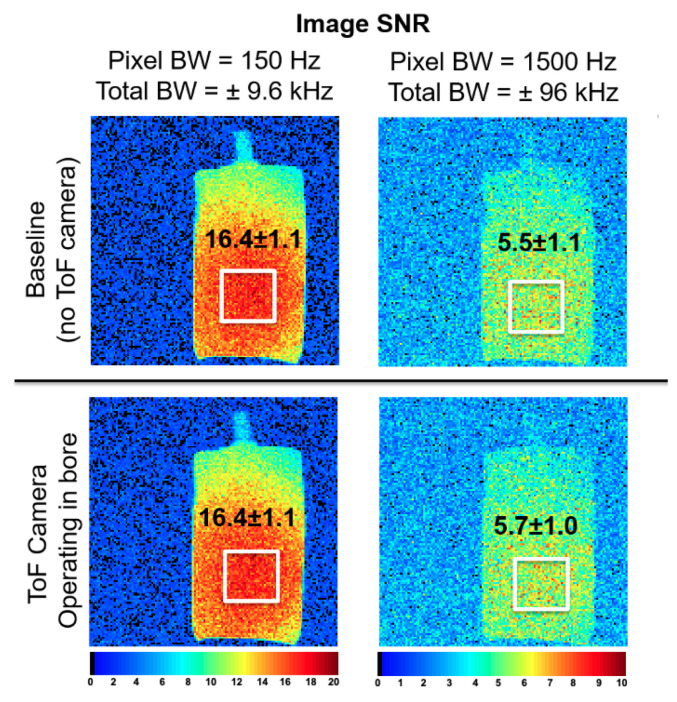
Effect of the Time-of-Flight (ToF) camera on MR image SNR. No statistically significant differences are observed between the image SNR with and without the ToF camera operational in the MR bore.

**Figure 7:**
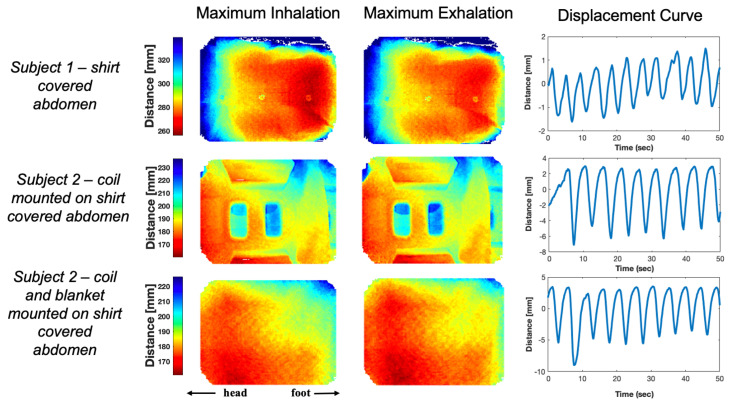
Time-of-Flight camera distance maps for subjects during maximum inspiration and expiration. (Top row) Subject 1 has their abdomen closest to the camera which experiences maximum motion during breathing. (Middle row) Subject 2 has their upper torso closest to the camera which experiences maximum motion during breathing and the camera is able to resolve distances even though there is a coil between the camera and the subject. (Bottom row) Subject 2, with a blanket placed on top of the body coil. The camera is able to resolve the distances with the blanket as well. In all three instances, averaging the camera *Z*−component over the entire region, the displacement curve shows a typical respiratory pattern.

Figure 6 shows the SNR images for baseline (no Time-of-Flight camera present in MR room) and during operation of the shielded Time-of-Flight camera. At a reasonable bandwidth (150 Hz/pixel), the average SNR in the rectangular ROI (Figure 6) is 16.4 +/− 1.1 without the camera operational, and the value remains unchanged with the camera operational. SNR maps with and without the camera at this bandwidth show no statistically significant difference (t-statistic = 0.70, *p*-value = 0.48, *alpha* = 0.05). At a large bandwidth (1500 Hz/pixel), the average SNR in the rectangular ROI (Figure 6) is 5.5 +/− 1.1 without the camera operational, and 5.7 +/− 1.1 with the camera operational. Once again, SNR maps show no statistically significant differences (t-statistic = 0.81, *p*-value = 0.41, *alpha* = 0.05). Therefore, the ToF camera has no adverse effect on image SNR, even at large receiver bandwidths that would be sensitive to interference over a wide range of frequencies. Figure 7 shows average distance to the abdomen of human subjects in the magnet during normal breathing as measured with the Time-of-Flight camera in different situations, including just the shirt-covered abdomen, the abdomen with a 6-channel flexible receive array positioned as for liver imaging, and also with a blanket on top of the abdomen and coil. In each case, the respiratory time course is clearly visible as the abdominal region rises and falls by several millimeters with each breath [28,29]. A time-course video of the ToF data acquisition can be found in the Supplementary Materials.

### 3.4. Outside the MRI: Scanner-Less Simultaneous Sensor Tracking of Respiratory Motion

**Figure 8:**
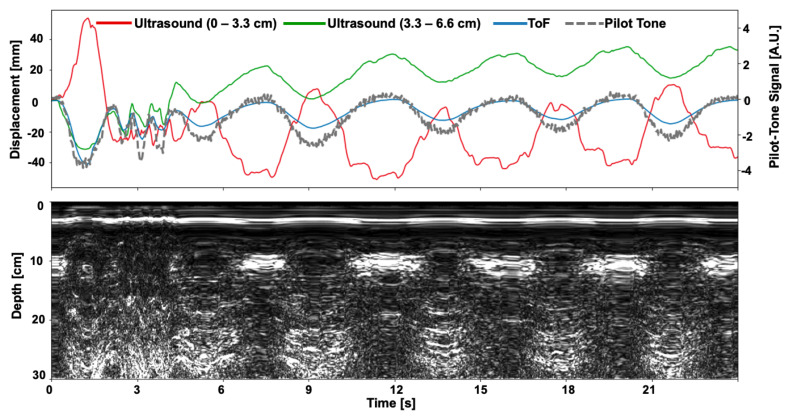
Simultaneous acquisition in all three sensors on a subject during respiration. The subject executed a deep breath followed by a series of coughs to assess the sensors’ responsiveness to these movements (top row). Over the depicted 25 s interval, all three sensors demonstrate a strong correlation in sensitivity to motion. Displacement curves derived from the Organ Configuration Motion (OCM) ultrasound sensor, captured at two different depths within the body, exhibit anti−phase motion behavior, indicative of organs moving in different directions relative to the ultrasound transducer. Pilot−Tone and Time−of−Flight (ToF) curves exhibit high correlation, with all sensors effectively capturing both deep breaths and coughs, also illustrated in the 2D ultrasound plot (bottom row).

Figure 8 shows the results of the simultaneous sensor experiment. Over a 25 s interval, all three sensors demonstrate a strong correlation in motion, throughout the deep breath and series of coughs in the first 4 s of the acquisition and the following period of normal respiration. In the top frame of Figure 8, displacement curves for ultrasound at two different depths and ToF are shown (mm, left axis) and a normalized signal curve for the Pilot-Tone is shown as well (arbitrary units, right axis). All three sensors show a good correlation in motion behavior. Compared to the other two sensors, the OCM sensor provides additional information, in that displacement curves derived from the ultrasound data, captured at two different depths within the body, exhibit out-of-phase behavior, indicative of reflective interfaces moving in different directions relative to the ultrasound transducer.

## 4. Discussion

In this study, we have shared preliminary experience for measuring displacement curves during 0.55 T MRI resulting from three different kinds of motion: liver motion resulting from respiration using the Pilot-Tone, bladder motion using the OCM ultrasound sensor, and anterior torso surface motion using the 3D ToF camera. For the Pilot-Tone and OCM sensor, we observed good correlations between the sensor signal and the observed motion and/or displacement as measured by the MR images. For the ToF camera, we were able to track surface motion during respiration. Additionally, our MR-less experiment showed the complementary and overlapping behavior of all three sensors, indicating that these sensors can all be used with or without the MR system, which has implications for use in therapy with correlation to previously acquired MR data [17].

For the Pilot-Tone experiment, to measure tissue-specific motion (of the liver, in this case), the ground-truth displacement is estimated directly in high-frame-rate MR images. In our application, a high level of correlation (almost linear) was found between the Pilot-Tone and liver displacement, warranting additional investigation on the generalizability of the Pilot-Tone when predicting the motion of other organs, while our proof of concept is demonstrated on the liver, in theory, the motion model development framework allows for the technique to be used in any organ or target tissue, as long as a fast imaging sequence can be used to extract the ground-truth motion. Additionally, we demonstrate our results using data exclusion beyond a pre-defined range of motion or when the volunteer is coughing. It should be noted, however, that our motion model enables the selection of any range of motion, which is effective in reducing motion artifacts (e.g., associated with deep inhalation/exhalation, or sudden bulk motion). Overall, the use of Pilot-Tone to compensate for respiratory as well as cardiac motion has been demonstrated before [14,15,30]. The Pilot-Tone has also shown a good correlation with ECG measurements for quantitative assessment of cardiac function [16]. Additionally, it has been used for myocardial T_1_ mapping, measurement of 5D flow-MRI, and respiratory correction [31,32,33,34]. The Pilot-Tone requires no contact with the subject being scanned, allowing for a non-cumbersome setup and ease of scanning. Ultimately, the Pilot-Tone is a diverse measurement tool, and validating its use with 0.55 T MRI or independent from the MR receive chain extends its range of potential applications.

The ultrasound experiments demonstrate that the OCM sensor can be used to detect motion in the bladder in addition to the measurement of respiratory and cardiac motion previously demonstrated [12,13]. Interestingly, the displacement curves obtained from different depths of the ultrasound data exhibit out-of-phase behavior. This was also observed in a previous study that employed these sensors to monitor respiratory motion [12], which could be indicative of ultrasound reflections from tissue interfaces that move in different directions relative to the ultrasound transducer during motion. In this study, we used a high-temporal-resolution MRI sequence to obtain lower abdomen images, however, in theory, a motion model could be trained to separate out the respiratory and internal organ motion as well. Similar to Pilot-Tone, the OCM sensor adds no additional acquisition time, unlike navigator sequences, while OCM sensors take a few minutes more to attach to the patient and set up compared to the respiratory belt, they are lightweight and can be used for multiple applications beyond motion correction due to their rich spatial information. They have been used to produce “scanner-less images” outside the MR bore by correlation to previously acquired MR data [17] and 4D MRI acquisition [35].

The use of a 3D Time-of-Flight (3D ToF) camera during MR imaging was demonstrated in this study. We demonstrated that for a wide range of acquisition bandwidths, MR sequences can be run with no measurable effect from the camera. Three-dimensional ToF has been used for assessment of subcutaneous tumor volume in an animal model [36] and tomography-based imaging techniques (such as CT) [37], but its use in MR has not been demonstrated previously. We anticipate that 3D ToF could be used for patient surface monitoring during radiation therapy. The use of 3D ToF for patient surface monitoring and characterization of respiration motion has been shown for 4D-CT [37], and it has also been used for calibration and set-up of multi-sensor positioning systems [38]. Similar to the Pilot-Tone, the 3D ToF does not require any subject contact and is, therefore, easy to set up. The presence of MR coils and blankets on the abdomen are not seen to inhibit the detection of the respiratory signal with the ToF camera. As we limited the application of the ToF camera to surface motion detection only, additional studies are required to see if the signal from the camera can be correlated with specific organ motions.

In a first-of-its-kind demonstration, we also acquired data from all three sensors simultaneously outside the MRI. Removing the dependency on MRI has important implications for applications such as live monitoring of motion during radiation therapy as this typically occurs outside the MRI bore, except in combined MRI–Linac systems [39]. In [39], the authors present a method to obtain motion-resolved 4D MRI for application in radiation therapy. This study highlights the need for motion tracking for therapy guidance, but self-navigation sequences still increase acquisition time, which is preferably short in radiation procedures. By augmenting MR data with pre-trained sensor data [17], motion tracking and correction techniques [11,39] may benefit from reduced acquisition times and/or additional information. The sensors used in this study demonstrate both complementary and overlapping behaviors. Additionally, the development of a “sensor suite” would enable training personalized, organ-specific motion models on each of the sensors, depending on their strengths. For example, our study, combined with others, indicates that the 3D ToF camera can be used to track surface body motion and the Pilot-Tone is useful for tracking superficial organ motion (heart, liver), whereas the OCM sensor is more versatile and can be used to potentially track superficial and deep organ motion. Our MR-less simultaneous sensor experiment shows that the ToF and Pilot-Tone sensors can both be calibrated using the ultrasound data, and vice versa. However, ultrasound would not work at bone interfaces or for surface body motion (since it is affixed to the surface) and would interfere with a beam path in case of radiation. Conversely, since the ultrasound and Pilot-Tone can be more difficult to interpret, the ToF can clearly indicate the difference between an inhalation and exhalation, since it measures the distance from a fixed point. In this way, there is a possibility to disentangle different motions that occur simultaneously (such as peristalsis and respiration) but are not related, by extracting only respiratory data from the ToF and internal motion from the ultrasound. Therefore, having a “sensor suite” would enable the user to select between the different sensors depending on their strengths and capabilities based on the use case.

Limitations of this study include the very small patient cohort. All of the sensors need to be tested and validated on larger cohorts with various pathologies at 0.55 T to both train more generalizable motion models and demonstrate their effective operation on unusual anatomies or obstructions due to tumors or variations in body morphology. In addition, while our initial proof-of-concept demonstration of motion correction using Pilot-Tone data uses a simple NUFFT reconstruction with motion-corrupted lines omitted, various more advanced motion-resolved reconstruction techniques are available and would be expected to yield improved results. Development (and validation in large patient cohorts) of techniques that are able to incorporate varying information from a full suite of sensors will be the subject of future work. Furthermore, the fast MR sequences used to calibrate the motion models have a very low spatial resolution, therefore, their capability to act as ground truth for obtaining motion curves for finer organs or structures needs to be further explored, while the performance of the Pilot-Tone has been previously compared to bellows and navigator trackers [14,18] and the ultrasound has been compared to optical tracking [12], we did not replicate such comparisons in this work, reserving detailed comparisons at 0.55 T for future work, while our Time-of-Flight camera experiments demonstrated the capability of this camera to show respiratory surface motion, we did not have simultaneous in vivo MR data to compare the ToF data to ground truth. However, its validation against the other sensors indicates its ability to similarly correlate to the motion of organs during respiration.

## 5. Conclusions

Three different sensors, a Pilot-Tone RF transmitter/receiver pair, an ultrasound sensor, and a 3D Time-of-Flight camera were used in this study. Their capability to elucidate motion of various kinds was demonstrated at 0.55 T, enabling motion tracking and motion correction. Additionally, the simultaneous use of all three sensors without the MR scanner was demonstrated, which may have interesting applications for radiation therapy guidance.

## Data Availability

The raw data supporting the conclusions of this article will be made available by the authors upon request.

## Supplementary Materials

The following supporting information can be downloaded at: https://www.mdpi.com/article/10.3390/s24123710/s1, Video S1: Realtime Time-of-Flight camera video.

## Abbreviations

The following abbreviations are used in this manuscript:

MRI	Magnetic Resonance Imaging
OCM	Organ Configuration Motion
PT	Pilot-Tone
RF	Radiofrequency
SNR	signal-to-noise ratio
ToF	Time-of-Flight
